# Observed behaviours and suicide assessment language post-Mental Health First Aid training in Australia and the United States: a mixed methods study using discourse analysis

**DOI:** 10.1186/s12909-022-03920-8

**Published:** 2022-12-05

**Authors:** William Nguyen, Rebekah Moles, Claire O’Reilly, Jennifer Robinson, Damianne Brand-Eubanks, Anne Kim, Jack C. Collins, Sarira El-Den

**Affiliations:** 1 The University of Sydney School of Pharmacy, Faculty of Medicine and Health, The University of Sydney, Camperdown, NSW Australia; 2 College of Pharmacy and Pharmaceutical Sciences, Washington State University, Spokane, WA USA

**Keywords:** Assessment, Discourse analysis, Mental health, Mental Health first aid, Student pharmacist, Suicide

## Abstract

**Background:**

Mental Health First Aid (MHFA) training teaches participants how to respond to mental health crises, including suicide. Little is known about the impact of training on participants’ observed MHFA behaviours. This exploratory study aimed to compare MHFA-trained Australian and US student pharmacists’ performance and suicide assessment language during simulated patient role-play (SPRP) assessments.

**Methods:**

Student pharmacists (*n* = 265) completed MHFA training and participated (*n* = 81) in SPRPs with simulated patients (SP) who were people with lived experience of mental illness. Each SPRP was marked by three raters (student, tutor and SP). One-way ANOVA, chi-squared tests and independent samples t-tests were used to compare scores and pass/fail rates, where appropriate. Transcribed audio-recordings of suicide assessments underwent discourse analysis. A chi-squared test was conducted to investigate the differences in how suicide assessment language was coded across six discursive frames (‘confident’/‘timid’, ‘empathetic’/‘apathetic’, and ‘direct’/‘indirect’).

**Results:**

Three raters assessed 81 SPRPs, resulting in quantitative analysis of 243 rubrics. There were no significant differences between student pharmacists’ mean scores and pass/fail rates across countries. Overall, both cohorts across Australia and the US performed better during the mania scenario, with a low failure rate of 13.9 and 19.0%, respectively. Most students in both countries passed their SPRP assessment; however, 27.8% did not assess for suicide or used indirect language during suicide assessment, despite completing MHFA training. Australian student pharmacists demonstrated, more direct language (76.9% versus 67.9%) and empathy (42.3% versus 32.1%) but less confidence (57.7% versus 60.7%) compared to US student pharmacists, during their suicide assessment; however, these differences were not statistically significant.

**Conclusions:**

Findings indicate most MHFA-trained student pharmacists from Australia and the US can provide MHFA during SPRPs, as well as assess for suicide directly, empathetically and confidently. This exploratory study demonstrates the importance of practicing skills post-training and the need for further research exploring participants’ hesitance to assess for suicide, despite training completion.

**Supplementary Information:**

The online version contains supplementary material available at 10.1186/s12909-022-03920-8.

## Background

Suicide is a serious global public health issue contributing to approximately 700,000 deaths annually yet, help-seeking behaviours and access to mental health services remain low due to barriers including stigma, discrimination from healthcare professionals and lack of awareness of effective treatments [1, 2]. Hom and colleagues’ review exploring mental health service use demonstrated a 29.5% service use rate among individuals who had ideated, planned, and/or attempted suicide in the previous year [3]. However, healthcare professionals often lack the fundamental knowledge and training that is needed to adequately recognise the signs of suicide and confidently intervene [4]. There has been increasing emphasis on developing and implementing suicide prevention training for frontline healthcare professionals and healthcare students, including current and future pharmacists [5–9]. For example, a broad range of healthcare professionals, including all licensed pharmacists practicing in Washington, are required to complete suicide prevention training [10]. Furthermore, training programs such as Mental Health First Aid (MHFA), which was originally developed for members of the public, have been used to educate primary healthcare professionals and healthcare students in caring for people experiencing mental health problems and crises, including suicide [11–13]. Specifically, the MHFA training program contains a component whereby participants learn about how to apply the MHFA Action Plan to assess for suicide risk and care for a person at risk of suicide [14]. MHFA training was developed in Australia and has now been adapted internationally and available in many countries including the US [15]. Morgan et al. assessed the effectiveness of MHFA training in a systematic review and meta-analysis whereby constructs such as participants’ knowledge, attitudes and self-reported behaviour in relation to people experiencing mental health problems and crises were found to significantly improve post-training [16].

Due to their high accessibility coupled with their expert knowledge in psychopharmacology, pharmacists regularly come into contact with people at risk of suicide [17]. This has been recognised by leading representative bodies, such as the Royal Pharmaceutical Society in the United Kingdom which has highlighted that pharmacists may have an expanding role in mental health if “they are trained as mental health champions and in Mental Health First Aid” [18]. The need for pharmacists to complete MHFA training has been recognised internationally, as evidenced by recent endorsement by the National Australian Pharmacy Students’ Association, and the American Pharmacists Association House of Delegates [19, 20].

The implementation of MHFA training amongst different degree programs, including pharmacy, has led to benefits such as improved mental health knowledge, self-reported behaviours and attitudes, as well as increased confidence relating to providing help for people experiencing mental health crises [13]. Yet, self-reported confidence in student pharmacists, post-MHFA training, does not necessarily translate to the ability to appropriately provide MHFA during observed assessments, with preliminary evidence demonstrating that some student pharmacists have difficulty using suicide-specific terminology and hesitate when inquiring about suicidal thoughts, despite having completed MHFA training [21]. This highlights that while MHFA training may have a positive impact on self-reported knowledge, attitudes and behaviours, its effect on actual or observed behaviours post-MHFA training, specifically with regard to the terminology and language used around suicide assessment, requires further research.

The manner by which healthcare professionals assess for suicide can impact how patients respond. For example, McCabe et al. investigated how healthcare professionals assess for suicidal ideation, demonstrating that over half of participating psychiatrists used a negative frame of questioning such as “*You don’t have thoughts of harming yourself?*” (p. 3), when assessing for suicide. Consequently, mental health consumers were more likely to respond that they were not having suicidal thoughts when assessed [22]. During MHFA training, participants learn about the importance of using direct language, as well as demonstrating empathy and confidence when assessing for risk of suicide; however, research exploring the language used by MHFAiders (i.e. people who have “completed a MHFA course delivered by an accredited MHFA Instructor”) when actually assessing for suicide risk is lacking.

MHFA training has been adopted into numerous pharmacy programs internationally, with a recent systematic review reporting that the majority of evaluation literature pertaining to MHFA training and assessment in university curricula is occurring in Australia and the US [13]. While there is a growing evidence base to support the effectiveness of MHFA training in improving self-reported constructs within student cohorts at specific universities, there is a lack of cross-cultural comparisons exploring the impact of MHFA training on student pharmacists’ helping behaviour and suicide assessment skills, specifically [23, 24]. Given that the MHFA training program contains specific content around how to assess and appropriately care for a person at risk of suicide, in conjunction with the increasing integration of MHFA training in pharmacy curricula, it is important to explore whether MHFA-trained student pharmacists are actually able to demonstrate these skills [12]. Having completed MHFA training, post-training, MHFAiders should be able to apply MHFA skills, including assessing and assisting people experiencing mental health crises [25]. Hence, the aim of this exploratory study was to compare Australian and US student pharmacists’ performance in simulated patient role-play (SPRP) assessments post-MHFA training. Specifically, this study was guided by the following research questions:

- Are there differences in how tutors, consumers and student pharmacists assess performance during SPRP?
- How do MHFA-trained Australian and US student pharmacists’ scores during SPRPs compare?
- What terminology do MHFA-trained Australian and US student pharmacists use to assess for suicide during SPRPs?
- Are there differences in how MHFA-trained Australian and US student pharmacists assess for suicide, with a focus on their confidence, empathy and use of direct language?

## Methods

### Study design

Student pharmacists studying in 2019 and 2020, who were in the final year of a four-year Bachelor of Pharmacy (BPharm) or a two-year Master of Pharmacy (MPharm) at The University of Sydney (USYD), as well as student pharmacists in the last 3 years of a six-year Doctor of Pharmacy (PharmD) from Washington State University (WSU) participated in this study. At USYD Pharmacy School, MHFA training is a compulsory component of the curriculum for both undergraduate and postgraduate student pharmacists who are enrolled in the final year of their degree. At WSU, MHFA training is offered as part of a two-credit mental health elective course. Following MHFA training, student pharmacists from Australia were assessed through SPRPs with mental health consumer educators recruited from One Door Mental Health [26]. Similarly, student pharmacists enrolled in the WSU MHFA elective were assessed through SPRPs with consumers and healthcare providers from the National Alliance on Mental Illness (NAMI) and Frontier Behavioural Health [27, 28]. Student pharmacists were randomly allocated to an SPRP assessment, whereby they had to provide MHFA to a mental health consumer/provider enacting one of three cases (Supplementary Material 1–3) as a SP. In both Australia and the US, each randomly selected student pharmacist had their SPRP observed by other fellow student pharmacists. Care was taken to ensure that the role-playing student pharmacist was not allocated to role-play a scenario that they had previously observed.

The SPRP assessments in both countries were audio-recorded. For each case, a case-specific grading rubric was marked out of a total score of 20 by a tutor to assess each student participant’s performance during the SPRPs. The same grading rubric was then given to the role-playing student pharmacist and the SP (consumer) immediately after the simulation, so they could also use it to self-assess and assess, respectively, the student’s performance in the SPRP. Hence, each student pharmacist was marked by three raters (student pharmacist self-assessment, tutor and consumer). While the three rater types (self-assessment, tutor and consumer) were consistent across all role-plays, different individuals contributed in each of these roles.

Student pharmacists were provided with an information sheet which explained the study conduct and purpose, and highlighted the voluntary nature of participation. It is important to note that these activities formed a compulsory part of curricula; however, student pharmacists could volunteer to consent to allow the research team to analyse their scores and audio-recorded assessments.

This study adopted a convergent parallel mixed method design. Between August 2020 to November 2020, data analysis was simultaneously and independently conducted once all student pharmacists’ scores and SPRP audio recordings were available from Australia and the US.

### Cases and rubrics

The development of the rubrics was guided by the MHFA Action Plan – ALGEE – along with a scoring system developed by MHFA researchers, and the rubrics have been used to assess student pharmacists participating in SPRPs post-MHFA training, supporting both face and content validity [14, 21, 29, 30]. The rubric has also been modified based on test re-test and interrater reliability analyses, to support its reliability across time and markers [31]. A score ranging from 0 to 2 was assigned to each item out of 10 items within the rubric (0 points = criteria not met, 1 point = some appropriate actions were demonstrated, 2 points = all appropriate actions were applied). To pass the case, student pharmacists needed to receive a score of at least 10 out of 20 and perform all the required actions.

The ‘required actions’ for each scenario are highlighted in grey within the respective grading rubrics, as can be seen in Supplementary Materials 1, 2 and 3. The required actions were all derived from concepts and skills taught during the MHFA training course in tandem with the MHFA manual and were agreed upon by the research team, all of whom were either MHFA instructors or MHFAiders [14]. As the SP admits to experiencing suicidal thoughts in the context of case 1, student pharmacists needed to ask the SP directly about suicidal thoughts, ensure that the SP was safe and not left alone as well as connect the SP with appropriate professional help, in order to pass the case (Supplementary Material 1). For case 2, student pharmacists were also required to assess the risk of suicide directly; however, the SP explains that they are not experiencing suicidal thoughts. Hence, a broader range of supports and professional help recommendations were appropriate for case 2, as the SP was not experiencing a mental health crisis (Supplementary Material 2). For case 3, student pharmacists were required to demonstrate that they ensured the SP received immediate professional help for an acute episode of mania (Supplementary Material 3). Hence, in all three cases, student pharmacists had to ensure the SP’s safety to pass.

### Analysis of SPRP scores across raters and countries

Quantitative analyses were conducted using IBM SPSS Statistics 27® (IBM Corp, Armonk, NY). Data used in the quantitative analyses was extracted from each student pharmacists’ rubric scores collected from each of the three raters. Additionally, pass/fail rates derived from the rubric scores of the tutor were used to compare student pharmacist performance between Australia and the US. A one-way analysis of variance (ANOVA) was used to determine the difference in means between the total rater scores by type of rater (student pharmacist, tutor and SP), independent of the case and country. Total rater score means from both countries were also compared using independent samples t-test to identify differences between Australian and US raters, independent of the case. Chi-squared tests were performed to compare pass/fail rates based on tutors’ scores from Australia and the US across cases 1–3. For all analyses, statistical significance was achieved when *p* ≤ 0.05.

### Discourse analysis

Each audio recording for cases 1 and 2 was transcribed verbatim for qualitative analysis as they were the cases that consisted of a mandatory suicide assessment criterion required to pass the SPRP assessment. A discourse analysis was performed to explore the language and terminology employed by student pharmacists when assessing for suicide. A discourse analysis is an analytical approach that involves deconstructing and critiquing language use, including its social context [32]. This approach was selected as it provided a critical outlook on the nature and form of the language and terminology used by each student pharmacist. Six discursive frames were developed a priori by four authors (WN, SE, RM and CO), adapted from MHFA guidelines as well as previous literature exploring how mental healthcare professionals communicate with patients about suicidal ideation [14, 22, 33]. During the suicide assessment, the student pharmacists’ dialogue was coded according to the following discursive frames: ‘confident’ or ‘timid’, ‘empathetic’ or ‘apathetic’, and ‘direct’ or ‘indirect’.

Student pharmacist dialogue was coded as ‘confident’ if they maintained composure and tone when speaking to the SP. On the other hand, student pharmacist dialogue was coded as ‘timid’ if they spoke with a shaky voice in conjunction with any delays in finishing words, prolonged hesitation, profuse stuttering, long pauses and having multiple disfluencies (e.g., “um…”, “like…”). Any long pauses or delays in speech which occurred during suicide assessment were transcribed and presented as an ellipsis (i.e., “…”). The ‘empathetic’ frame reflected whether student pharmacists displayed a genuine sense of care for the SP while they assessed for suicide. The statements used immediately before, during and after the suicide assessment were used to determine whether student pharmacist dialogue was coded as either ‘empathetic’ or ‘apathetic’. Language considered to be ‘direct’ during suicide assessment was characterised by referring to suicide explicitly as well as using recommended direct terminology, as per MHFA guidance, for example, using terms such as ‘suicide’, ‘killing yourself’ or ‘suicidal thoughts’ [14]. Student pharmacists were coded as using an ‘indirect’ approach to assessing for suicide if they used ambiguous language and terminology that is either obscure, vague and/or convoluted. For example, questions that incorporated words such as ‘harming yourself’, ‘injuring yourself’, ‘negative thoughts’ or ‘dark thoughts’ demonstrated an ‘indirect’ suicide assessment. If a student pharmacist combined direct and indirect terminology (e.g., “Are you thinking of harming yourself or suicide?”) within the same sentence, the dialogue was coded as ‘indirect’. Student pharmacists were considered ‘direct’ if the questions were asked separately (e.g., “Are you thinking about self-harm?”, “Are you thinking about suicide?”) and enough time was allocated for a response from the SP in between each question, in that it was evident that they were asking about thoughts of both non-suicidal and suicidal self-injury.

A decision to not include a ‘neutral’ option as a discursive frame was made by three authors (WN, SE and RM) as discussion between the authors made it possible to reach consensus and code the data within the dichotomous frames. Despite having been introduced as a frame within some previous discourse analysis methodologies, a ‘neutral’ frame is not always used in studies specifically exploring the language used in suicide assessment [22, 33, 34]. Moreover, a ‘neutral’ frame was considered to have been inappropriate if applied to the discursive frames within this study. For example, with regard to the ‘direct’ or ‘indirect’ frames, what constitutes direct assessment of suicide is clearly articulated in the MHFA manual [14].

One coder (WN) listened to each recording in full and extracted key data relating to the timing of the suicide assessment, the specific terminology used in relation to suicide, as well as coded each suicide assessment as per the aforementioned discursive frames. A second coder (RM) then listened to the specific excerpt of the audio-recordings when the suicide assessment took place and independently coded each student pharmacist’s suicide assessment using the six discursive frames, blinded to the coding of the first coder (WN). To determine the level of inter-rater agreement between the two coders, Cohen’s Kappa values were calculated using IBM SPSS Statistics 28.0. A comparison of the coders’ individual assessments was conducted for each student pharmacist participant, whereby any discrepancies in coding were replayed in a meeting with a third co-author (SE) and discussed until agreement by consensus was reached by three co-authors (WN, RM and SE).

### Cross-country comparison of discourse analysis

A chi-squared test was conducted to determine whether there were any significant differences between participants from Australia and the US regarding the proportion of suicide assessments coded across the discursive frames. Independent samples t-tests were performed to determine whether there were any significant differences in the duration of the SPRPs and the timing when suicide assessment occurred, between Australian and US data.

### Considerations relating to authors’ reflexivity

WN, SE and RM led the analyses and made the core decisions regarding the discursive frames. SE is an accredited MHFA instructor, registered pharmacist and educator. RM is MHFA-trained and has had over 2 decades of experience in community pharmacy practice and as an academic educator. SE and RM were part of the team that led the development of the first simulation assessments, and they have both taken part in these assessments since. Both SE and RM have played the role of the tutor as well as the simulated patient, in previous assessments. Their contribution was influenced by their knowledge of MHFA guidance, as well as their experience of community pharmacy practice and of assessing student pharmacists during simulated patient assessments. At the time of data analysis, WN was a MHFA-trained final year student pharmacist undertaking his honours research project. He had first-hand experience with supporting consumers living with mental illness in his role as a student working in community pharmacy.

## Results

**Fig. 1:**
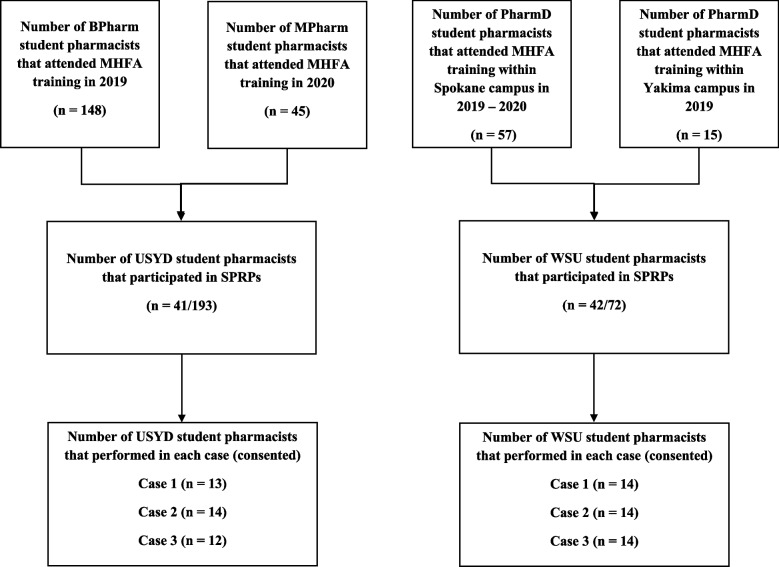
Flow diagram of student pharmacists who completed Mental Health First Aid (MHFA) training and Simulated Patient Role-Play (SPRP) assessments

A total of 265 student pharmacists participated in this study. In Australia, 148 student pharmacists from the Bachelor of Pharmacy (BPharm) in 2019 and 45 student pharmacists from the Master of Pharmacy in 2020 participated. Among the 193 student pharmacists from USYD, 41 were allocated to role-play as the MHFAider in the SPRP, as part of their curriculum. At WSU, 72 second- and third-year student pharmacists undertook an elective unit called ‘Mental Health First Aid’. Student pharmacists were recruited from two campuses of WSU – Spokane (*n* = 57) and Yakima (*n* = 15), of which 42 student pharmacists were allocated to role-play the MHFAider in the SPRP, as part of their curriculum in the Fall 2019 and Spring 2020 terms. In total, 39/41 student pharmacists from Australia and 42/42 from the US (Fig. 1) consented to the use of their data for research.

### Comparison of overall rater mean scores independent of case and country

Of the 81 student pharmacists who participated in SPRPs, each student pharmacist was scored by three different raters, which resulted in 243 rubrics being analysed quantitatively. The overall mean score was lowest among self-assessments conducted by student pharmacists, followed by tutors then SPs. The ANOVA analysis (Table 1) demonstrated a statistically significant difference in total mean scores between raters, independent of the case and country (F[2240] = 7.60, *p* = 0.001). A Tukey post-hoc test revealed that there was a significant difference between the mean scores of the student pharmacist group and the SP (consumer) group (*p* < 0.001), as well as the student pharmacist group and the tutor group (*p* = 0.048).

**Table 1:** Comparison of overall rater mean scores independent of case and country

							df	
Rater	Overall Mean Score	SD	SE	95% CI	***p***-value	F	Between groups	Within groups
Tutor	15.7	3.3	0.4	15.0 to 16.4	0.001	7.60	2	240
SP (Consumer)	16.5	3.2	0.4	15.8 to 17.2				
Student Pharmacist	14.5	3.0	0.3	13.9 to 15.2				

### Comparison of rater mean scores between countries independent of cases

The independent samples t-tests (Table 2) demonstrated no significant differences between the mean scores of any rater types across countries (*p* > 0.05).

**Table 2:** Comparison of rater mean scores between countries independent of cases

Rater	Country	N	Overall Mean (Out of 20)	SD	Independent samples t-test
Tutor	USA	42	15.9	3.8	t[79] = 0.60, *p* = 0.55
	Australia	39	15.5	2.7	
SP (Consumer)	USA	42	16.1	3.2	t[79] = −0.92, *p* = 0.36
	Australia	39	16.8	3.1	
Student Pharmacist	USA	42	14.8	3.2	t[79] = −0.93, *p* = 0.36
	Australia	39	14.2	2.9	

### Comparison of tutor pass/fail rates across all cases between countries

Student pharmacists from the US who had completed case 1 for their SPRP assessments achieved the highest failure rate with 40.5% of student pharmacists receiving a fail grade for their performance, while the lowest failure rate (13.9%) was seen in Australian student pharmacists participating in case 3. Results from the chi-squared test in Table 3 demonstrate no statistically significant difference between Australian and US student pharmacist pass/fail rates for each case (*p* > 0.05).

**Table 3:** Comparison of tutor pass/fail rates for each case

				Pearson Chi-Square	
Case no.	Country	% that passed SPRP assessment (%)	% that failed SPRP assessment (%)	Value	df
1	USA	59.5	40.5	0.44	1
	Australia	66.7	33.3		
2	USA	78.6	21.4	0.07	1
	Australia	76.2	23.8		
3	USA	81.0	19.0	0.37	1
	Australia	86.1	13.9		

### Discourse analysis findings

Fifty-five student pharmacists across both countries participated in cases 1 and 2 which involved assessing the SP for suicide risk. One recording was found to be missing due to a technical error and could not be analysed; therefore, 54 audio-recordings were available for analysis; 26 (Case 1 = 12, Case 2 = 14) from Australia and 28 from the US (Case 1 = 14, Case 2 = 14). There was an equal number (*n* = 4) of student pharmacists from both countries who did not assess for suicide risk during their SPRPs; hence, eight recordings (14.8%) did not undergo discourse analysis, rendering 46 valid cases for analysis. Examples of illustrative quotes extracted from the audio-recorded SPRPs used to inform the coding of each of the discursive frames are shown in Table 4.

**Table 4:** Illustrative quotes of discursive frames

Frame	Student pharmacist quote
Confident	“Have you thought about suicide?” [Student no. 18]
Timid	“Considered it? Um…okay um…I think you should…um you should contact the primary care doctor and um…and talk more with the primary care doctor…and um you could also talk to your sister especially um…make sure you get the help. Um…would you like me to call um…” [Student no. 22]
Empathetic	“It seems like there might be something else going on. Um I’m a little concerned when you mentioned that you’re feeling hopeless and I don’t want you to take this the wrong way but I really care about you and your well-being so I wanna know” [Student no. 1]
Apathetic	“Oh okay. That doesn’t sound good. Make sure um…do you have anyone else at home or are you living by yourself at the moment?” [Student no. 37]
Direct	“Are you experiencing any suicidal thoughts or have you thought about suicide?” [Student no. 21]
Indirect	“Have you been thinking of um harming yourself or doing something?” [Student no. 36]

Coding of ‘direct vs indirect’ language used by student pharmacists had the greatest level of inter-rater agreement (κ = 0.563 (95% CI = 0.238–0.888; *p* < 0.001), whereby the Cohen’s Kappa value demonstrated a “moderate” level of agreement between the coders [35]. There was less agreement among coders in coding ‘empathetic or apathetic’ frames as indicated by a “fair” level of agreement between the coders (κ = 0.253 (95% CI = 0.09–0.416); *p* = 0.006) [35]. The least level of agreement among coders was in relation to coding the ‘confident or timid’ frames, as indicated by in a “slight” level of agreement (κ = 0.132 (95% CI = − 0.142-0.406); *p* = 0.320) [35]. In total, the two coders disagreed on 40 frames across a total of 28 recordings, whereby all 28 suicide assessment recordings were replayed in the presence of both coders and a third co-author (SE), and discussed until agreement by consensus was reached by the three co-authors (WN, RM and SE). The final coding results are presented in Supplementary Material 4.

### Quantitative comparison of discourse analysis and audio-recordings

Australian student pharmacists used more direct language (76.9% versus 67.9%) and displayed more empathy (42.3% versus 32.1%) than US student pharmacists during their suicide assessment, but were found to be slightly less confident (57.7% versus 60.7%). However, a chi-squared test revealed no statistically significant differences in the proportions of student pharmacists from each country categorised into each of the discursive frames (*p* > 0.05).

Independent samples t-tests demonstrated that the average total duration for the SPRPs in the US (4.3 mins) was significantly shorter than in Australia (7.8 mins), (t[44] = − 3.52, *p* < 0.001). US student pharmacists assessed for suicide risk significantly earlier into the SPRP (at 2.1 mins) than Australian student pharmacists (at 3.9 mins) (t[44] = − 5.75, *p* < 0.001) (Table 5).

**Table 5:** Comparison of the total duration of SPRPs and timing of suicide assessment between countries

Dependent variable	Country	N	Mean time (minutes)	SD (minutes)	Independent samples t-test
Time of suicide assessment	USA	24	2.1	1.4	t[44] = −5.75, *p* = 0.001
	Australia	22	3.9	2.1	
Total duration of SPRP	USA	24	4.3	1.8	t[44] = −3.52, *p* < 0.001
	Australia	22	7.8	2.3	

## Discussion

This study is among the first to explore how MHFA participants across two countries apply their knowledge and skills gained from MHFA training in observed behavioural assessments, thereby expanding the current evidence base which is heavily reliant on self-reported measures of knowledge, attitudes and behaviours post-MHFA training. MHFA training is increasingly embedded in healthcare curricula, and there have been calls in both Australia and the US to make the training a requirement for pharmacists [13, 19, 20]. Systematic reviews have demonstrated the effectiveness of MHFA training in improving self-reported confidence and behaviours; hence, the current exploratory study evaluating observed behaviours post-training provides an important addition to the literature [16, 36]. The quantitative results demonstrated that there were no statistically significant differences between MHFA participants’, namely student pharmacists’, performance during observed behavioural assessments, as evidenced by cross-country comparisons of pass/fail rates and raters’ mean scores. Furthermore, the majority of student pharmacists passed each of the three cases across both countries, indicating their ability to apply the MHFA Action Plan when assessed across a range of mental health problems and crises. Hence, these findings may be the first to demonstrate that performance post-MHFA training is similar among training participants, internationally, regardless of the version of the training taught (i.e. Australian versus US version). Nonetheless, current findings were derived from post-training evaluations only and experimental studies are needed to further explore and confirm the results. Findings also demonstrate that post-MHFA training the majority of participants were able to use direct terminology to appropriately assess for crisis, as evidenced by 76.9% of Australian and 67.9% of US student pharmacists coded as “direct” during the discourse analysis. However, this indicates that approximately 23% of Australian student pharmacists and 32% of US student pharmacists did not use direct language in their suicide assessment despite completing MHFA training. Furthermore, less than 50% of student pharmacists from both countries were coded as ‘empathetic’ when assessing for suicide. Hence, further education in this area, and opportunities to practice suicide assessment skills in an empathetic manner may be needed for student pharmacists. While exploratory, this study is the first to explore cross-country applications of MHFA through objective, observed assessments requiring demonstration of actual behaviours, and starts to build the evidence in this area.

### Student pharmacists’ performance in SPRPs

As presented in Table 3, 33.3% of Australian student pharmacists and 40.5% of US student pharmacists failed case 1, while 23.8% of Australian student pharmacists and 21.4% of US student pharmacists failed case 2. Case 3, which depicted a SP with mania symptoms but no suicidal thoughts, had the lowest failure rate with 19.0 and 13.9% of student pharmacists from Australia and US, respectively, failing the SPRP assessment. Interestingly, student pharmacists had marked themselves more harshly as demonstrated in Table 1, as this was the lowest rater mean score independent of country. This finding is contradictory to other pharmacy literature which conveyed that student pharmacists’ self-assessment marks were higher when compared to marks provided by SPs and faculty instructors during a communication assessment [37]. On the other hand, Langendyk reported that high-achieving, 3rd-year medical students marked themselves and their peers more harshly and with relatively higher accuracy when compared to the faculty’s mark. Furthermore, students who achieved low marks marked themselves more generously than the faculty which may have been attributed to students’ lack of self-assessment skills to adequately assess competence [38]. Multiple factors including the context in which self-assessment is occurring could affect how students assess their performance. For example, Evans et al. found that dental students in oral surgery had felt stressed knowing that their self-assessment was being observed or evaluated [39]. Hence, it is possible that student pharmacists from USYD and WSU may have experienced pressure causing them to underscore themselves given that they were assessed in the same room as other peers, tutors and SPs.

### Student pharmacists’ use of direct language and terminology during suicide assessment

MHFA participants are taught to use direct language when assessing a person for risk of suicide [14]. Eight student pharmacists did not assess for suicide when providing MHFA to the SP in cases 1 and 2. Of the 46 participants who did assess for suicide, five US and two Australian student pharmacists used indirect language (Supplementary Material 4), indicating that 15 student pharmacists (27.8%) overall either did not assess for suicide or assessed for suicide using indirect language. Similarly, Wilson et al. explored US student pharmacists’ performance in a simulated counselling assessment post-suicide prevention training and found that although student pharmacists self-reported improved confidence in assessing for suicide directly, factors such as stigmatising thoughts, apprehension towards the topic of suicide and biased perspectives acted as barriers to student pharmacists asking about suicide during the assessment [24]. El-Den et al. have also explored Australian student pharmacists’ MHFA skills in SPRPs post-MHFA training and similarly found that 40.0% of participants used ambiguous terms or avoided suicide-specific terminology completely when assessing for suicide during SPRPs with tutors [21]. Furthermore, O’Reilly et al. explored how mental healthcare professionals question at-risk youth about self-harm and suicide and found that participating youth had not been routinely asked questions directly during suicide risk assessment [40]. Possible reasons for reluctance to ask about suicide directly may be self-perceived incompetence, fear or discomfort as a result of working with individuals at risk of suicide or believing that suicidal thoughts may be induced if suicide-specific terminology is used [41, 42]. Future research exploring MHFAiders’ and healthcare professionals’ views on the use of direct language when assessing for suicide is warranted.

### Cross-country comparisons of suicide assessments

As presented in Table 5, the duration and time taken to assess for suicide between Australian and US student pharmacists differed significantly (*p* ≤ 0.001), whereby Australian student pharmacists took approximately twice the amount of time in total, as well as to arrive at the point in the SPRPs where they enquired about suicide, as compared to US student pharmacists. Despite being 8 hours in duration, MHFA USA covers the same content areas as the 12-hour Australian MHFA course [43, 44]. A potential reason for the difference between the total duration of SPRPs and timing of suicide assessment could be that during MHFA training in Australia, student pharmacists may have possibly more time dedicated to role-plays and practicing active listening and using open-ended questions. Another theory as to why the total duration of SPRPs was significantly different may be that student pharmacists from the US pre-emptively knew that their SPRPs would involve a mental health case scenario as the student pharmacists were enrolled in a mental health elective course. In Australia, however, student pharmacists participate in SPRPs which test their ability to perform a range of first-aid skills of which one is MHFA; hence, they may not immediately recognise that the case relates to a mental health problem or crisis. Further, cultural factors may be responsible for the observed differences. Bell described differences in relation to suicide, based on cultural standpoints within a country [45]. For example, historically within the Japanese culture, suicide is considered to be an ‘honourable’ act as a means for appeasing an individual’s public disgrace while suicide is seen as a ‘sinful’ act within certain Islamic countries [45]. Future research focusing on cross-cultural comparisons of suicide assessment, as well as preferences in relation to length of time for suicide-related discussions is needed.

### Student pharmacists’ empathy during suicide assessment

During MHFA training, student pharmacists are taught to non-judgmentally and empathetically communicate with people experiencing mental health problems and crises [14]. However, the results of the discourse analysis in this study suggested that a higher proportion of student pharmacists displayed apathy rather than empathy during the SPRPs. The broader literature surrounding teaching and assessing empathy among healthcare students highlights the need for further high quality, robust research in this area, as well as a consistent definition of “empathy” across studies. There is evidence to suggest that among medical students empathy decreases as they progress throughout their degree, yet, contrastingly, among student pharmacists there may be significant increases in empathy scores as student pharmacists progress across the year levels of the degree [46, 47]. However, these findings vary across settings, with variations to this phenomenon reported across different studies [48]. Nonetheless, simulation activities have been shown to improve empathy among student pharmacists and medical students, and various other effective interventions have been identified to improve empathy [46, 49, 50]. Empathy is often a difficult concept to teach and assess, and while students may be able to appreciate the importance of empathy, they may not always be able to actually demonstrate empathy when listening and communicating with others, particularly if they have not been provided with opportunities to practice and reflect on their empathy skills in safe, learning environments [51]. Demonstrating apathy when communicating with patients can impact patient-provider relationships and health outcomes. For example, apathy towards people living with mental illness may result in decreased disclosure of suicide-related problems, symptoms and behaviours, as well as perceiving healthcare professionals as hostile, uncaring or insincere [52]. There is limited research exploring the impact of MHFA and post-training assessments on empathy, specifically; however, research is emerging in this area. A recent study exploring the impact of MHFA training on student pharmacists’ empathy demonstrated significant increases in empathy post-training, highlighting the importance of the training in enhancing communication skills [53]. Hence, further research evaluating the impact of MHFA training on a broad range of participant populations’ ability to demonstrate empathy, as well as the sustainability of changes is warranted.

### Strengths and limitations

This is the first study comparing MHFA participants’ performance in post-training observed, behavioural assessments across countries. Nonetheless, several limitations need to be considered, when interpreting the findings. Firstly, it should be noted that the current study evaluated performance post-MHFA training, only, emphasising the need for future research employing other study designs (e.g. pre−/post-studies) to further investigate the impact of MHFA training on participants’ provision of MHFA during SPRPs.

Secondly, as opposed to MHFA training which is compulsory for all final year student pharmacists at USYD, the decision to enrol in an elective course including MHFA training was based on the student pharmacists’ own choice at WSU. Thus, a potential limitation could be that student pharmacists from the US were more willing and engaged than the Australian cohort as they volunteered to undertake the elective course; however, the lack of significant differences in scores demonstrate that this is unlikely. Furthermore, while care was taken to ensure no role-playing student was allocated to role-play a case that they had already observed, it is possible that the scores of those who were randomly allocated to roleplay a case after they had already observed a different case were impacted and this should be considered when interpreting the findings. However, a study that compared student pharmacists’ confidence after completing MHFA training and participating in the simulated assessments as a role-playing or observing student pharmacist demonstrated similar improvements in self-reported confidence [29]. Nonetheless, future research with larger sample sizes exploring the effect of being an observer on student performance during future observed behavioural assessments, across a range of scenarios, is warranted.

Finally, another potential limitation was that the authors who contributed to the discourse analysis were all from Australia; thus, each audio-recording was analysed through an Australian lens. It is important to note that the results from the discourse analysis may possibly be perceived differently based on the nationality of the coder, as cultural differences may affect how different individuals analyse and interpret the same discourse, as identified by Bouton [54]. However, a strength of the method employed was the use of two independent coders and the presence of a third co-author to assist in resolving disagreements by consensus. Nonetheless, the “slight” to “moderate” levels of agreement as indicated by the Cohen’s Kappa values demonstrate the need for further research and clear guidance on the conduct of discourse analysis relating to suicide assessment, which is an area of research that is emerging and especially important given suicide is ‘serious public health problem’ which is largely preventable [2, 35]. The authors acknowledge the exploratory nature of the current study and the need to interpret findings with caution. Recommendations from study findings include the need to conduct further research, with larger MHFAider sample sizes and multiple coders to confirm current findings and to build the evidence base in this area. Furthermore, the discourse analysis may have been strengthened by using video recording to allow for analysis of non-verbal communication, and future studies with ethics approval to analyse video-recordings are needed.

## Conclusions

This study provides a unique exploration and comparison of Australian and US student pharmacists’ performance in SPRPs of mental health scenarios post-MHFA training, to start to address the gaps in the evidence base surrounding observed assessment of MHFA provision, lack of cross-country comparisons of MHFA training and language surrounding suicide assessment. Discourse analysis demonstrated no significant differences in terms of student pharmacists’ ability to demonstrate confidence, empathy and the use of direct language when assessing for suicide, in Australia and the US. However, despite receiving adequate training, and consistent with findings from other studies, some student pharmacists in both countries still assessed for suicide using indirect language or did not assess for suicide at all, despite suicide risk factors and warning signs within scenarios. Therefore, there is a need for research exploring reasons for reluctance to use direct terminology and how to best teach and assess empathy among current and future healthcare professionals, as well as MHFA participants generally. Given that MHFA is now widely available internationally, further evaluation of training, including in-depth evaluations of the language used when assessing for suicide and cross-country comparisons of actual behaviours across diverse participant populations is warranted.

## Supplementary Information

**Additional file 1.**

## Data Availability

The data is not publicly available due to privacy or ethical restrictions.

## Abbreviations

MHFA: Mental Health First Aid
US: United States
SPRP: Simulated Patient Role-Play
SP: Simulated Patient
MPharm: Master of Pharmacy
USYD: The University of Sydney
PharmD: Doctor of Pharmacy
WSU: Washington State University
GP: General practitioner
PRE: Pre-suicide assessment
POST: Post-suicide assessment
SA: Suicide assessment
