# Supplementary material for: Observed behaviours and suicide assessment language post-Mental Health First Aid training in Australia and the United States: a mixed methods study using discourse analysis
Source: BMC Med Educ. 2022 Dec 5;22:838. doi: 10.1186/s12909-022-03920-8 (PMC9720991; doi:10.1186/s12909-022-03920-8)
Supplement: Supplementary file 1 — Additional file 1. [file 12909_2022_3920_MOESM1_ESM.docx]

**Supplementary Material**

**Supplementary Material 1**. Case 1 MHFA SPRP Assessment Information and Grading Rubric

**Case 1 - anxiety/depressive symptoms with suicidal thoughts**

**OPENING LINE FOR EDUCATOR**

Tina/Tim

“Hi, I’m feeling quite tired lately, since my divorce, and would like your advice regarding a multivitamin for energy”.

**INFORMATION FOR SIMULATED PATIENT**

You are very flat and lethargic in nature. Since the divorce you have no energy and can’t even get out of bed in the morning. You have little motivation to go to work and don’t have energy to go anywhere else. You are therefore looking for a vitamin to help with energy levels.

**Key phrases to mention: “**I just feel like it’s not worth it anymore’ and that ‘there is no point in life”

**Possible Questions**

| How long have you been feeling this way? | Since divorce, 3 months |
| --- | --- |
| Have you told anyone how you are feeling? | Sister worries about them |
| Have you sought professional help? | No, don’t want to bother them |
| Have you had thoughts of suicide?  *[students should ask about this]* | Considered it |
| Have you ever attempted suicide before? | No |
| Do you have a plan if you were to attempt suicide?  *[students should ask about this]* | Sometimes I think I should just drink all the alcohol I have at home and swallow a box of 100 panadol pills. Or sometimes I look at the balcony and consider jumping after the wine. |
| Have you been using alcohol or drugs recently? | Yes, alcohol (a bottle of wine a night) more recently. It takes the feelings away |
| Have you had mental illness/depression before? | Yes I had depression in the past |
| Who is the personal contact? | Sister |
| Phone number to contact you on? | 0447 887 342 (made up number) |
| Are you happy for me to call professional help for you? | No, but sister will take me straight to the GP to talk about it.  If student insists and plan is reasonable (e.g. lets walk over to GP or call crisis number together here, then patient agrees. |

Notes: potential depressive episode, triggered by negative, personal events, recently

**Scoring Guide:**

- Actions shaded in GREY must be performed by the participant to pass the case.
- The participant must receive a score of at least 10/20 AND perform all actions shaded in GREY to pass the case.
- *Phrases and actions* that are *italicized* are merely suggestions and examples, and do not need to be articulated by the participant word-for-word.
- Phrases and actions that are underlined must be performed by the participant, to pass the item.

| **Item** | **ACTION** | **FULL MARKS (2)** | **PARTIAL MARKS**  **(1)** | **NO MARKS (0)** |
| --- | --- | --- | --- | --- |
| **1** | **Approaches consumer appropriately**   - *Introduces self* - *Greets consumer* |  |  |  |
| **2** | **Provides comfortable setting for consumer to talk**   - *Negotiates privacy* - *Ensures the consumer is comfortable* |  |  |  |
| **3** | **Listens and communicates non-judgmentally**   - *Does not blame the consumer* - *Does not use words like “alcoholic”, “druggie”* - *Non-judgemental attitudes displayed* |  |  |  |
| **4** | **Asks appropriate open-ended questions**   - *How can I help?* - *How long have you been feeling this way?* - *What type of support have you sought?* - *How are you coping?* - *Who have you spoken to about your emotions?* |  |  |  |
| **5** | **Asks if the consumer is having suicidal thoughts**   - Asks directly (*e.g. Are you thinking of killing yourself/ending your life/suicide?).*   If the participant asks using indirect language, only, (e.g. harming/hurting yourself), then only give partial marks (1).  *Participant MUST assess for suicide risk to pass the case. |  |  |  |
| **6** | **Asks appropriate follow-up questions in relation to consumer’s suicidal thoughts**   1. *Do they have a plan? (e.g. how/when they will suicide)* 2. *Have they attempted suicide before?* 3. *Are they using alcohol/drugs?*   Note: participants MUST enquire about a “plan”, otherwise “0” marks for this item.  If the participant asks about the plan, only, give one partial mark.  If the participant asks about the plan, and at least one other question, give the full two marks. |  |  |  |
| **7** | **Gives reassurance and appropriate information**   - *They care and want to help* - *Thoughts of suicide are often associated with treatable mental illness* - *Thoughts of suicide are common and do not have to be acted on* - *Mental illness is common* - *Effective psychological/pharmacological treatment for mental illness exists* |  |  |  |
| **8** | **Displays empathy**   - *Uses empathetic language (e.g. “I can see that this is a difficult time for you”)* - *Does not use sympathetic language (e.g. “I feel sorry for you”)* - *Demonstrates continual willingness to help* |  |  |  |
| **9** | **Takes appropriate action**   1. Does not leave the consumer alone (*e.g. connect with family member/friend to pick up, calls Mental Health Crisis Team/ambulance to pick up*) 2. Connects the consumer with appropriate professional help (*e.g. Calling Mental Health Crisis Team/Ambulance, Lifeline/Suicide call back service or immediately see the doctor*)   *Participant MUST do both of these actions to pass the case and receive full marks (2) for this item.  Additional examples of follow-up actions   - *Takes phone number to call for follow up* - *Provides pharmacy number for consumer to call back* |  |  |  |
| **10** | **Good non-verbal communication**   - *open body language* - *appropriate eye contact* - *non-judgemental facial expressions* |  |  |  |
| **TOTAL (out of 20)** | |  | | |

**Supplementary Material 2**. Case 2 MHFA SPRP Assessment Information and Grading Rubric

**Case 2 – anxiety/depressive symptoms with no suicidal thoughts**

**OPENING LINE FOR EDUCATOR**

Laura/Logan

“Hi, I’m having trouble falling asleep lately, and would like your advice regarding

sleeping tablet for insomnia. I have a 10 month old baby at home and taking care of him this past year has completed ruined my sleeping cycle!”

**INFORMATION FOR EDUCATOR**

You are very flat and lethargic in nature. You are constantly tired, you can’t sleep, you have not attended your regular parents’ group in months. You were supposed to go back to full-time work at 6 months but have taken a further 6 months of leave because you just aren’t feeling up to it. Husband/wife is great but off at work 8am-7pm, and travels a lot for work, so you feel bad burdening him/her when she/he works so hard.

**Key phrases to mention: “**It’s just too hard’, ‘I don’t know if I can keep doing this’

**Possible Questions**

| How long have you been feeling this way? | about 6 months |
| --- | --- |
| Have you told anyone how you are feeling? | Mother and father worries about them |
| Have you sought professional help? | No, don’t have time |
| Have you had thoughts of suicide?  *[students should ask about this]* | NO |
| Have you ever attempted suicide before? | NO |
| Do you have a plan if you were to attempt suicide? | NO |
| Have you been using alcohol or drugs recently? | Yes, 1-2 glasses of wine a night |
| Have you had depression before? | Had panic attacks as a teenager but not recently |
| Who is the personal contact? | husband/wife |
| Phone number to contact you on? | 0447 887 342 (made up number) |
| Are you happy for me to call professional help for you? | Yes ok. An in house visit from GP would be good  I will try and get back into mothers/fathers groups – they were helpful and fun |

**Scoring Guide:**

- Actions shaded in GREY must be performed by the participant to pass the case.
- The participant must receive a score of at least 10/20 AND perform all actions shaded in GREY to pass the case.
- *Phrases and actions* that are *italicized* are merely suggestions and examples, and do not need to be articulated by the participant word-for-word.

| **Item** | **ACTION** | **FULL MARKS (2)** | **PARTIAL MARKS**  **(1)** | **NO MARKS (0)** |
| --- | --- | --- | --- | --- |
| **1** | **Approaches consumer appropriately**   - *Introduces self* - *Greets consumer* |  |  |  |
| **2** | **Provides comfortable setting for consumer to talk**   - *Negotiates privacy* - *Ensures the consumer is comfortable* |  |  |  |
| **3** | **Listens and communicates non-judgmentally**   - *Does not blame the consumer* - *Does not use words like “alcoholic”, “druggie”* - *Non-judgemental attitudes displayed* |  |  |  |
| **4** | **Asks appropriate open-ended questions**   - *E.g. “How can I help?”* - *E.g. “How long have you been feeling this way?”* - *E.g. “What type of support have you sought?”* - *E.g. “How are you coping?”* - *E.g. “Who have you spoken to about your emotions?”* |  |  |  |
| **5** | **Asks if the consumer is having suicidal thoughts**   - Asks directly (*e.g. “Are you thinking of killing yourself/ending your life/suicide?”).*   If the participant asks using indirect language, only, (*e.g. harming/hurting yourself*), then only give partial marks (1).  *Participant MUST assess for suicide risk to pass the case. |  |  |  |
| **6** | **Gives reassurance and appropriate information**   - *They care and want to help* - *Mental illness is common* - *Effective psychological/pharmacological treatment for mental illness exists* |  |  |  |
| **7** | **Displays empathy**   - *Uses empathetic language (e.g. “I can see that this is a difficult time for you”)* - *Does not use sympathetic language (e.g. “I feel sorry for you”)* - *Demonstrates continual willingness to help* |  |  |  |
| **8** | **Takes appropriate action**   - *Offers a range of professional services (e.g. GP, Beyondblue, Black Dog Institute, psychologist, psychiatrist)* - *Encourages discussing with family member/friend/partner* - *Takes phone number to call for follow up* - *Provides pharmacy number for consumer to call back* |  |  |  |
| **9** | **Encourages self-help**   - *Lifestyle recommendations (e.g. exercise, eating well)* - *Relevant support groups (e.g. widowers, carers, divorcees, new mothers)* - *E.g. “Have you told anyone else about this?”* |  |  |  |
| **10** | **Good non-verbal communication**   - *Open body language* - *Appropriate eye contact* - *Non-judgemental facial expressions* |  |  |  |
| **TOTAL (out of 20)** | |  | | |

**Supplementary Material 3**. Case 3 MHFA SPRP Assessment Information and Grading Rubric

**Case 3 – mania symptoms with no suicidal thoughts**

**OPENING LINE FOR EDUCATOR**

Jake/Jackie

“Hi, I’m here to pick up a refill for my antidepressant. It has been working really well. I have really noticed a difference in the last week.”

**INFORMATION FOR EDUCATOR**

This is a prescription that you filled for the first time a little over a month ago and this is the first time you are refilling the prescription. You are very hyper and can’t seem to maintain a conversation without jumping from subject to subject but state “you have never felt better, I have so much energy”. You have called into work sick a couple of times this week but feel it is fine as the company would shut down if you didn’t work there. You have not had to take your medications everyday as they seem to work when you need them.

**Key Phrases to mention**: “I have so many ideas it is hard to keep track” and these meds really worked fast” and “I don’t have to sleep much anymore with all this energy”

**Possible Questions**

| How long have you been feeling this way? | 2 maybe 3 days |
| --- | --- |
| Have you told anyone how you are feeling? | No, I don’t see the doctor for another month |
| Have you sought professional help? | No, I have all this energy and ideas, other people are just having trouble keeping up. |
| Have you had thoughts of suicide?  *[students may ask about this, not the focus of the case]* | No, that is why I went to the doctor last month. I feel great now. |
| Have you ever attempted suicide before?  *[students may ask about this, not the focus of the case]* | No |
| Do you have a plan if you were to attempt suicide?  *[students may ask about this, not the focus of the case]* | No, haven’t thought about it seriously |
| Have you been using alcohol or drugs recently? | Yes, alcohol seems to slow me down enough to get a little sleep. Six-pack in the evening |
| Have you had mental illness/depression before? | Yes, that is why I was put on the citalopram |
| Who is the personal contact? | My brother but we had a falling out yesterday because he was jealous of my intelligence. |
| Phone number to contact you? | 509-123-4567 |
| Would you allow me to discuss these changes in mood with your provider and help you set an appointment? | I don’t know, I think I am doing great. I think I could probably provide help to my doctor. |

Notes: Potential manic episode that may be inappropriately treated or caused by the antidepressant

**Scoring Guide:**

- Actions shaded in GREY must be performed by the participant to pass the case.
- The participant must receive a score of at least 10/20 AND perform all actions shaded in GREY to pass the case.
- *Phrases and actions* that are *italicized* are merely suggestions and examples, and do not need to be articulated by the participant word-for-word.

| **Item** | **ACTION** | **FULL MARKS (2)** | **PARTIAL MARKS**  **(1)** | **NO MARKS (0)** |
| --- | --- | --- | --- | --- |
| **1** | **Approaches consumer appropriately**   - *Introduces self* - *Greets consumer* |  |  |  |
| **2** | **Provides comfortable setting for consumer to talk**   - *Negotiates privacy* - *Ensures the consumer is comfortable* |  |  |  |
| **3** | **Listens and communicates non-judgmentally**   - *Does not blame the consumer* - *Does not use words like “alcoholic”, “druggie”* - *Non-judgemental attitudes displayed* |  |  |  |
| **4** | **Asks appropriate open-ended questions**   - *E.g. “How can I help?”* - *E.g. “How long have you been feeling this way?”* - *E.g. “What type of support have you sought?”* - *E.g. “How are you coping?”* - *E.g. “Who have you spoken to about your emotions?”* |  |  |  |
| **5** | **Conducts appropriate assessment**   - *Recognise signs & symptoms of possible panic attack, manic episode or severe psychotic state* - *Assesses urgency of current situation* |  |  |  |
| **6** | **Gives reassurance and appropriate information**   - *They care and want to help* - *If panic attack – e.g. provide reassurance (they are safe & symptoms will pass, empathise with distress)* - *If psychoses – e.g. empathise with distress, symptoms are real* |  |  |  |
| **7** | **Displays empathy**   - *Uses empathetic language (e.g. “You seem very upset”)* - *Does not use sympathetic language (e.g. “I feel sorry for you”)* - *Demonstrates continual willingness to help* |  |  |  |
| **8** | **Takes appropriate action**  **For example:**   - *worsening mania/psychosis needs immediate referral*   *OR*   - *first-time panic attack requires immediate, emergency care*   *Other appropriate actions*   - *Offers a range of professional services (e.g. GP, case manager, psychologist, psychiatrist, hospital emergency department or calling ambulance)* - *Encourages discussing with family member/friend/partner* - *Takes phone number to call for follow up* - *Provides pharmacy number for consumer to call back* |  |  |  |
| **9** | **Encourages self-help**   - *Lifestyle recommendations (e.g. exercise, eating well)* - *Relevant support groups (e.g. widowers, carers, divorcees, mental health support groups)* - *E.g. “Have you told anyone else about this?”* |  |  |  |
| **10** | **Good non-verbal communication**   - *Open body language* - *Appropriate eye contact* - *Non-judgemental facial expressions* |  |  |  |
| **TOTAL (out of 20)** | |  | | |

**Supplementary Material 4**. Discourse analysis of Australian and US student pharmacists’ suicide assessments (n = 54)

|  |  | **Discursive frames** | | |
| --- | --- | --- | --- | --- |
| **No.** | **Student Pharmacist Quote(s)** | **Confident or Timid** | **Empathetic or Apathetic** | **Direct or Indirect** |
| **USA** | | | | |
| **1** | **PRE** *– It seems like there might be something else going on. Um I’m a little concerned when you mentioned that you’re feeling hopeless and I don’t want you to take this the wrong way but I really care about you and your well-being so I wanna know*  **SA** *– if you’ve been having thoughts of suicide?*  **POST** *– I’m really sorry to hear that, that’s a really really hard thing to go through.* | Confident | Empathic | Direct |
| **2** | **PRE** *– None*  **SA** *– Are you thinking about suicide or harming yourself?*  **POST** *– You have considered it? Would it be okay if I shared some resources with you about suicide and prevention? And…you know surprisingly, a lot of people go what you’re feeling. You know a lot of people go through this and I can’t totally understand how you feel but I’m here to help you.* | Confident | Apathetic | Direct |
| **3** | **PRE** *– So I know like you’re um going through a really tough time right now, you’re really sad. So I have to ask*  **SA** *– like have you been thinking about like possibly hurting yourself or like committing suicide?*  **POST** *– Okay, yeah so I know that for whatever you’re going through, it seems like you’re in like a bottomless pit, you’re never going to get out of it but I know that as long as you keep trying to get help, keep talking to people, keep reaching out, eventually you’ll get out of it.* | Confident | Apathetic | Indirect |
| **4** | **PRE** *– None*  **SA** *– Have you actually thought of maybe having thoughts of suicide at all or anything in that result?*  **POST** *– Have you considered it? Has that been recent?* | Confident | Empathetic | Direct |
| **5** | **PRE** *– Have you…um I have another question for you, if you feel comfortable answering it.*  **SA** *– Um have you ever thought about hurting yourself?*  **POST** *– You have? Okay. Yeah so I actually have some phone numbers that I’d like to give you before I want to talk to you um about the energy levels and stuff like that.* | Confident | Apathetic | Indirect |
| **6** | **PRE** *– It does seem like it's a lot to handle.*  **SA** *– Have you um…ever then kind of had that feeling of either hopelessness or um…just wanting to you know either um like harm yourself, harm somebody else? Have you had any of those thoughts?*  **POST** *– No? Okay.* | Confident | Apathetic | Indirect |
| **7** | **PRE** *– Okay…well this is just a question, um just cause I want to make sure but*  **SA** *– have you had any thoughts of suicide?*  **POST** *– Okay…well that’s good to hear. Um have you tried any other um…mood boosting things in the past? That helped? Or things that helped you to…get excited for?* | Timid | Apathetic | Direct |
| **8** | **PRE** *– None*  **SA** *– None*  **POST** *– None* | N/A | N/A | N/A |
| **9** | **PRE** *– None*  **SA** *– Okay um have you considered um killing yourself lately?*  **POST** *– Okay, so I’m a little concerned cause it sounds like you have depression and I would consider asking a um calling a provider to enquire about this?* | Confident | Empathetic | Direct |
| **10** | **PRE** *– …And I understand that is a lot of work that you’re doing and it definitely take a toll on your mental health as well, um…have you thought about like self-harm?*  **SA** *– Or have you had any like suicidal thoughts lately?*  **POST** *– Yeah…well that’s good to hear.* | Timid | Empathic | Indirect |
| **11** | **PRE** *– Um…have you had any um thoughts about harming yourself?*  **SA** *– How about thoughts about killing yourself?*  **POST** *– Okay, um do you live alone right now?* | Confident | Apathetic | Direct |
| **12** | **PRE** *– None*  **SA** *– None*  **POST** *– None* | N/A | N/A | N/A |
| **13** | **PRE** *– But I’m concerned about you. I know that some days are hard to get out of bed and go along with your day-to-day life, um are you feeling like you might be depressed?*  **SA** *– Or have you had thoughts of suicide?*  **POST** *– And what kind of things have you been considering? Is it something that you’ve potentially thought could bring to life?* | Confident | Empathetic | Direct |
| **14** | **PRE** *– Do you feel like you’re…um…so when I hear you say like you can’t take it anymore, I feel like I get the feeling like you’re really…um you’re maybe sad or depressed about this. Is that correct?*  **SA** *– Um have you ever…thought about like taking your own life or anything?*  **POST** *– No? Okay. That’s good to hear.* | Timid | Empathetic | Direct |
| **15** | **PRE** *– There are things that I can you know give to you to help with the sleeping um but I’m just a little bit more concerned with how you’re doing with the situation as you don’t seem well. You seem, you tell me that you’re at your wits’ end, you tell me you can’t handle this anymore. I just wanted to make sure, if I can*  **SA** *– have you ever thought about you know suicide or attempting suicide?*  **POST** *– Well thank you for letting me know, I just wanted to make sure because you’re well-being is my main concern.* | Confident | Empathetic | Direct |
| **16** | **PRE** *– Okay, have you ever thought of harming yourself?*  **SA** *– Any suicidal thoughts?*  **POST** *– No? Ok, so I think you might have postpartum depression. That’s what it sounds like. A lot of pregnant women will feel that after they have the baby cause it’s a lot, like a big burden on you and it’s a lot of like responsibility and sometimes you might feel like it’s overwhelming.* | Confident | Apathetic | Direct |
| **17** | **PRE** *– Okay um*  **SA** *– are you considering suicide?*  **POST** *– Okay, thank you for telling me.* | Confident | Apathetic | Direct |
| **18** | **PRE** *– None*  **SA** *– Have you thought about suicide?*  **POST** *– Do you have a plan?* | Confident | Apathetic | Direct |
| **19** | **PRE** *– None*  **SA** *– Are you having thoughts of suicide?*  **POST** *– You have, okay. So I think it’s really important that you talk with the doctor um cause there are medications that can help with that definitively, they can help with um your fatigue as well as your mood. Um…do you have any support systems around you right now? Like family, friends?* | Confident | Apathetic | Direct |
| **20** | **PRE** *– None*  **SA** *– Are you…thinking about killing yourself?*  **POST** *– Okay, and…how else are you feeling right now?* | Timid | Apathetic | Direct |
| **21** | **PRE** *– Okay.*  **SA** *– Are you experiencing any suicidal thoughts or have you thought about suicide?*  **POST** *– You’ve considered it? Okay, have you talked with anyone about it? Any family members?* | Timid | Apathetic | Direct |
| **22** | **PRE** *– …*  **SA** *– Are you feeling suicidal?*  **POST** *– Considered it? Um…okay um…I think you should…um you should contact the primary care doctor and um…and talk more with the primary care doctor…and um you could also talk to your sister especially um…make sure you get the help. Um…would you like me to call um…* | Timid | Apathetic | Direct |
| **23** | **PRE** *– None*  **SA** *– None*  **POST** *– None* | N/A | N/A | N/A |
| **24** | **PRE** *– None*  **SA** *– None*  **POST** *– None* | N/A | N/A | N/A |
| **25** | **PRE** *– Sorry about that.*  **SA** *– Are you um having thoughts of suicide?*  **POST** *– Okay, have you spoken to your doctor about this yet?* | Confident | Apathetic | Direct |
| **26** | **PRE** *– So I really want to know um*  **SA** *– are you having thoughts of suicide?*  **POST** *– Yeah well that is a relief to hear from me. Um should that ever change, I want you to know that you can always come back to me as a resource and um we have a list of um hotlines and social support groups that you can utilise and they really have a good trained staff. Um but I think for now the most important thing we can do for you is to discuss with your primary care physician to see if we can um get you something that will help you to fall asleep at night.* | Confident | Empathetic | Direct |
| **27** | **PRE** *– For your um…sleep cyc…like…is there anything else like besides sleep that you’re thinking about like*  **SA** *– do we have any um…thoughts of like suicide or anything like that?*  **POST** *– And you’re doing great, you’re just getting help right now to just help get that sleep cycle all set out. Um…* | Timid | Apathetic | Indirect |
| **28** | **PRE** *– But there are times where you know that these can be tough and that’s perfectly understandable that what you’re going through. Um I just want to know if like after 9 months of feeling this way*  **SA** *– have you thought about um killing yourself?*  **POST** *– Um are there any excess medications that are out in the house?* | Confident | Empathetic | Direct |
| ***Australia*** | | | | |
| **29** | **PRE** *– Um have you been having any other thoughts at all?*  **SA** *– Any thoughts of suicide or anything like that?*  **POST** *– No? Okay.* | Confident | Empathetic | Direct |
| **30** | **PRE** *– Um look um…this sometimes um…people react to situations differently. Um but can I just ask. Just to be straight.*  **SA** *– have you had any thoughts about suicide?*  **POST** *– Okay, um that’s good news obviously. Um but um what I think might be going on here is um we like to call it medically as postpartum depression um it’s just a way of um identifying…* | Confident | Empathetic | Direct |
| **31** | **PRE** *– Sorry finally um…just one more question,*  **SA** *– do you ever feel suicidal or anything?*  **POST** *– Okay.* | Confident | Apathetic | Direct |
| **32** | **PRE** *– None*  **SA** *– None*  **POST** *– None* | N/A | N/A | N/A |
| **33** | **PRE** *– None*  **SA** *– None*  **POST** *– None* | N/A | N/A | N/A |
| **34** | **PRE** *– Okay I see um…*  **SA** *– have you um…have you had any suicidal…thoughts or…behaviour or…situation so far?*  **POST** *– …Actually I think so many Australians have some depression but I think many Australians are…saved by professional support so I really recommend you um once see a doctor or seek professional support…um at this stage do you think you need something help? Um professional help?* | Timid | Apathetic | Direct |
| **35** | **PRE** *– I'm really sorry to hear that. Motivation okay in that case. And given that you've told me that you had a past history of depression. Um if you don't mind me asking,*  **SA** *– have you had any thoughts of suicide lately? Or…*  **POST** *– Oh that's not good to hear. Um…I'm sorry that you have those um thoughts um especially going through a divorce. It's not that easy um…if I may ask, um have you um…made a plan regarding those thoughts? Or made any intentions of acting upon those thoughts?* | Confident | Empathetic | Direct |
| **36** | **PRE** *– Um…I'm willing to hear what um you want to tell about your story. I’m happy to hear whatever you want to say um so…regarding these*  **SA** *– have you been thinking of um harming yourself or doing something?*  **POST** *– You haven’t really planned anything?...okay um…I really appreciate that you take the courage to share your feelings. Um…in my opinion I do recommend you talking to, um getting support from um people around you, for example um getting some appropriate professional advice because um you mentioned that you did have the suicide thought.* | Timid | Empathetic | Indirect |
| **37** | **PRE** *– No um it’s actually a good idea to visit a GP and to see what they can um do to help you with your condition. Um don’t think about um like you bothering them because I’m sure they’re always happy to like help you whenever you need. Um I'm just wondering,*  **SA** *– do you have any suicidal thoughts or anything like that?*  **POST** *– Oh okay. That doesn't sound good. Make sure um…do you have anyone else at home or are you living by yourself at the moment?* | Confident | Empathetic | Direct |
| **38** | **PRE** *– All good. Thank you for sharing me with that. I just want to ask,*  **SA** *– are you thinking about suicide in any possible way?*  **POST** *– And have you made any plans for this?* | Confident | Apathetic | Direct |
| **39** | **PRE** *– Yeah okay um…so you said before that you've been thinking that it's not worth it anymore?*  **SA** *– Have you been thinking about um taking your own life?*  **POST** *– Have you had any kind of thoughts about a plan to do it or did you have anything to kind of act upon that? Have you made any preparation or anything?* | Confident | Empathetic | Direct |
| **40** | **PRE** *– I'm going to um ask you a question which is um can be very confronting and I would appreciate if you will be honest with me.*  **SA** *– Um have you ever contemplated or thought about um taking your life or suicide or something?*  **POST** *– And have you planned how you want to approach the situation?…Okay, okay. Fair enough.* | Confident | Apathetic | Direct |
| **41** | **PRE** *– …Okay…*  **SA** *– Um have you ever thought about suicide or anything like that?*  ***POST*** *– …and have you had any plans or have you tried it?* | Timid | Apathetic | Direct |
| **42** | **PRE** *– Okay yep…um…okay so I’m going to ask a question that might be a bit direct. Um so have you intended on suicide?*  **SA** *– Have you thought about suicide?*  **POST** *– Okay. So you just feel overwhelmed?* | Timid | Apathetic | Direct |
| **43** | **PRE** *– So um…just regarding…just cause um cause you said that you’re very um…feeling a bit down recently. I just want to ask a question because I’m quite concerned about you.*  **SA** *– Have you ever had thoughts about suicide in the past or…recently?*  **POST** *– I see yeah um…do you um…so you don’t um…have you ever told anyone else about how you’ve been feeling?* | Timid | Empathetic | Direct |
| **44** | **PRE** *– None*  **SA** *– None*  **POST** *– None* | N/A | N/A | N/A |
| **45** | **PRE** *– So you said that you don’t know how long you can keep doing this. Um do you plan on not doing this for long? Have you been having any plans of escaping?*  **SA** *– Okay, so you haven’t had any suicidal thoughts have you?*  **POST** *– Okay good.* | Confident | Apathetic | Indirect |
| **46** | **PRE** *– None*  **SA** *– None*  **POST** *– None* | N/A | N/A | N/A |
| **47** | **PRE** *– Um you can see your GP and then you get a referral for um psychologist or psychiatrist um it depends. Um but let me ask you a question first um…*  **SA** *– have you had any direct thoughts about suicide?*  **POST** *– I know it's quite hard and frustrating but um trust me everything um in life, every problem has a solution. You just need to work on it. Um it will take a little bit of time.* | Confident | Apathetic | Direct |
| **48** | **PRE** *– So can I ask you, you said that things aren’t um…you know you don’t see much of a point in things anymore,*  **SA** *– have you considered um suicide? Have you considered um killing yourself recently?*  **POST** *– Yeah. Well I would really like to help you work through these things so I can give you some advice and um recommend some services if you like. And um I'm always here to talk.* | Confident | Empathetic | Direct |
| **49** | **PRE** *– Okay so do you mind if I ask*  **SA** *– if you have any suicidal thoughts?*  **POST** *– How many times like do you have that idea for?* | Confident | Apathetic | Direct |
| **50** | **PRE** *– Um I mean a lot of people feel like that quite often. Um I know it can be difficult especially with what you’ve been through. Um so there are some options that we can um discuss but I'll just ask um*  **SA** *– have you considered taking your own life?*  **POST** *– It sounds like you're a bit depressed at the moment.* | Confident | Empathetic | Direct |
| **51** | **PRE** *– Um do you mind if I ask*  **SA** *– have you any suicidal thoughts?*  **POST** *– Yep. So I was wondering, just wanted to make sure.* | Confident | Apathetic | Direct |
| **52** | **PRE** *– Um sometimes we get so much pressure from our surroundings and environment and it can really affect our mental health. Um it happens to a lot of people that I meet and there’s always um…we can always seek help with medical advisors. Um so I know just because it’s a mental health um problem doesn’t mean there’s anything wrong with you, it just means that sometimes we’re very tired and then we can be pressured a lot and it makes us feel very tired. So because um yeah so I feel like that’s one thing that can be a problem, have you had…I just want to ask you a question um*  **SA** *– have you had any thoughts of suicide lately?*  **POST** *– That’s okay, can I ask if you are taking any other medications?* | Confident | Empathetic | Direct |
| **53** | **PRE** *– Yeah um…I can help you with that but just with your situation, I am a little bit concerned um…may I ask um*  **SA** *– have you ever thought of suicide?*  **POST** *– Um…do you talk to anyone about…do you have a trusted family doctor that you talk to about your…* | Timid | Empathetic | Direct |
| **54** | **PRE** *– Yeah um…I do have a question I have to ask first um,*  **SA** *– have you ever had any thoughts on suicide?*  **POST** *– Okay…so um…how is your insomnia?* | Timid | Apathetic | Direct |

**Abbreviations**

GP = General practitioner, PRE = Pre-suicide assessment, POST = Post-suicide assessment, SA = Suicide assessment
